# Here I Am, Despite Myself

**DOI:** 10.1371/journal.ppat.1005106

**Published:** 2015-09-10

**Authors:** Glenn Rall

**Affiliations:** Fox Chase Cancer Center, Philadelphia, Pennsylvania, United States of America

As I sit down to write this abbreviated tale of my laboratory’s research and my own professional journey, I’m struck by how things have turned out. I often will tell students who visit Fox Chase Cancer Center, where I’ve been a faculty member for 20 years, that a common misconception about those of us who have been research scientists for some time is that ours was an intentional journey: that perhaps I knew from an early age that I would be a viral neuroimmunologist, and that each step along the way was thoughtfully considered and deliberate.

Hardly.

I was born in 1963, and thus I was an impressionable 6-year-old when Apollo 11 landed on the moon. I could see the moon from my backyard in New Jersey—vast, oddly glowing, and wildly mysterious—and the idea that humans had walked on it was, in the truest sense of the word, incredible. Thus, Career Choice #1 was to be an astronaut; I began the preparation for this life choice by insisting on bedsheets adorned with planets and stars, and Tang to drink with every meal. The ideations of a 6-year-old faded, eventually. In high school, I earned some extra money by being a kids’ birthday party magician—I was actually pretty good at it—and thus Career Choice #2 was to be an illusionist (which sounded more serious than “magician”). Long-term success in this arena was more realistic than space travel, but only marginally so. When I went off to college, I had “come back to Earth” with my career plans and decided I would make a good pediatrician, but life (OK, organic chemistry) intervened to thwart Career Choice #3. By chance, as this light also faded, a door opened to work in a research lab studying acid rain and bacterial populations, which I accepted. I’d never considered research science as a vocation, but I found that I loved the open-endedness of the process; unlike a magic trick, the outcome was not preordained. Thus, after graduation, I went off to graduate school, initially to be a bacteriologist, but eventually working at Vanderbilt University with Tamar Ben-Porat, a pioneer in herpesvirus biology and pathogenesis. Five years later, with my PhD in hand, I moved to San Diego for a fellowship with Michael Oldstone at the Scripps Research Institute, and came to Philly in 1995, where I’ve been on the faculty at Fox Chase ever since.

Despite this apparently Brownian career path, I’m certain that my considered careers, no matter how transient, influenced how I approach which projects we pursue. That is, these apparent dead-end career decisions were actually instrumental in how my lab approaches science.

Our lab works at the intersection of virology, immunology, and neurobiology, studying viral movement in neurons and antiviral immunity in the brain using mouse models and primary neuron cultures. One of the viruses that we use most extensively is measles virus, long known for its ability to rapidly kill the cells it infects. But soon after beginning our studies with measles virus infection of neurons, we realized that neuronal viral spread was fundamentally different from the classical spread described in textbooks for other cell types: measles virus resulted in a nonlytic infection in neurons without release of infectious progeny. This led us to propose the hypothesis (certainly conceived of by others long before us) that the “viral particle” and the “infectious particle” are distinct entities. The “virus particle” is the virion itself: the genome and associated viral proteins that are released from an infected cell. But the “infectious particle” is the virion plus the requisite cellular proteins needed to complete the viral life cycle. As obligate intracellular parasites, viruses must co-opt cellular proteins to perform most steps in reproduction; as such, various cell types (with distinct profiles of cellular proteins) offer different “tool kits” to the virus, which may radically alter how the virus reproduces. We believe that the nature of the neuron, which relies on coordinated trafficking of macromolecules to and from the synapse, fosters movement of the virus along the axon to favor nonlytic, trans-synaptic spread, rather than the typical lytic pathway.

Neurons also differ in fundamental ways in their response to the antiviral host response. While a key antiviral cytokine, interferon gamma, is needed for resolution of a neuronal viral infection, as it is for many peripheral infections, the signaling pathways within the neuron (that is, how the interferon “signal” is interpreted) is wholly distinct. In fact, Stat1, a key signaling molecule associated with interferon gamma, is not required at all for neuronal protection. So, once again, a response is tailored based on the nature of the affected cell. And again, the notion promulgated in textbooks and reviews is only partly correct, and rarely accounts for the influence of cell type.

Throwing spitballs at dogma has been wonderful fun, but it has been sobering as well. There is comfort in telling ourselves that we actually understand a biological process, and when data appear that complicate or challenge our simplistic view, it is easy to wonder how deep this complexity actually goes. There are occasions when I wonder if the human intellect actually has the capacity to fully understand all of life’s mysteries: for example, how memories are retained and recalled, how diverse organs work in concert, or how cellular responses to an external cue are interpreted. But what a grand purpose and great privilege to dedicate one’s professional career to tackling these Big Questions and, like the astronaut I once wished to be, to venture into the unknown, confident that marvels await.

**Image 1:**
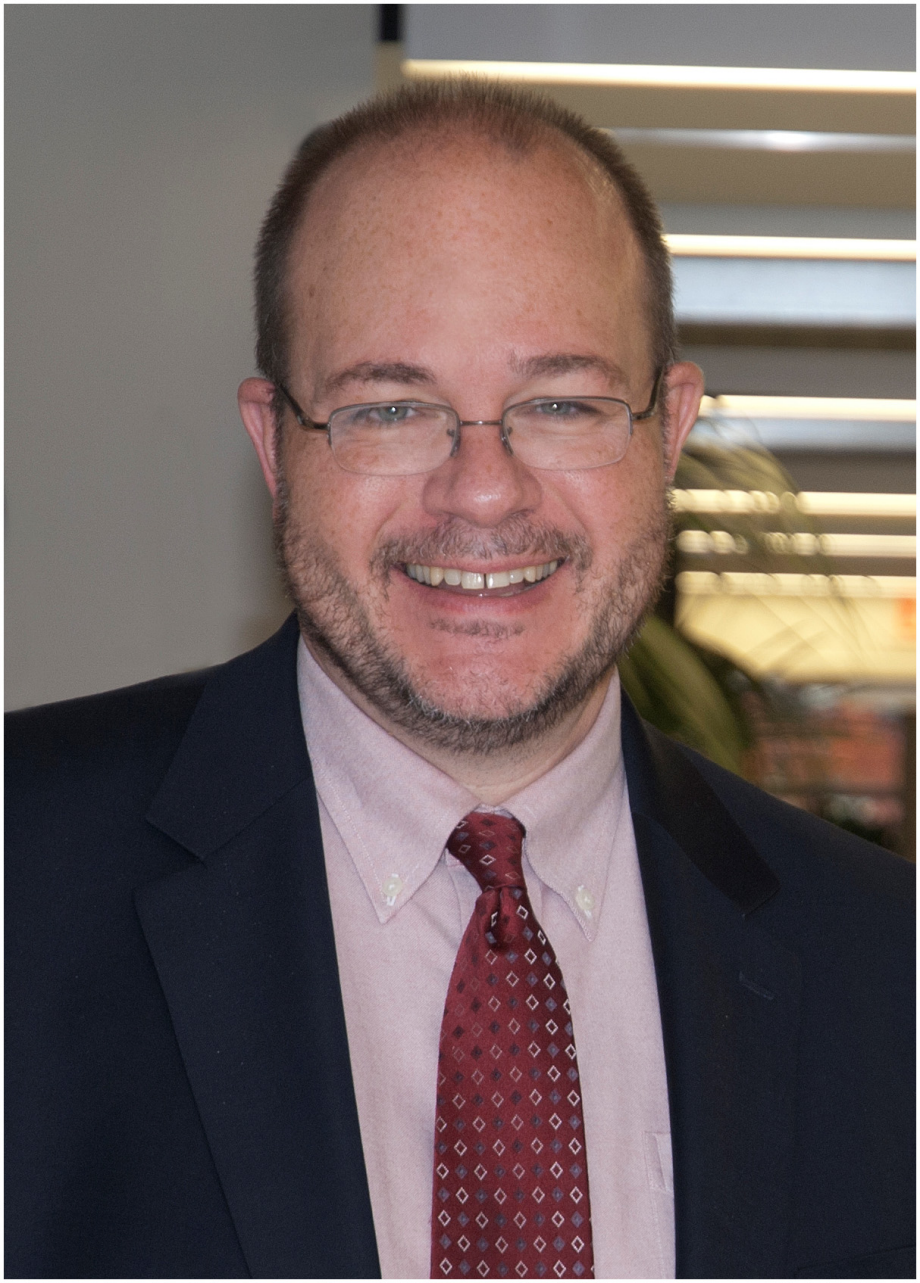
Glenn Rall.

